# TIM-3/Gal-9 interaction affects glucose and lipid metabolism in acute myeloid leukemia cell lines

**DOI:** 10.3389/fimmu.2023.1267578

**Published:** 2023-11-10

**Authors:** Mahnaz Rezaei, Mustafa Ghanadian, Behrooz Ghezelbash, Abolfazl Shokouhi, Alexandr V. Bazhin, Andrey A. Zamyatnin, Mazdak Ganjalikhani-Hakemi

**Affiliations:** ^1^ Department of Immunology, Faculty of Medicine, Isfahan University of Medical Sciences, Isfahan, Iran; ^2^ Department of Pharmacognosy, School of Pharmacy, Isfahan University of Medical Sciences, Isfahan, Iran; ^3^ Endocrine and Metabolism Research Center, Isfahan University of Medical Sciences, Isfahan, Iran; ^4^ Department of General, Visceral and Transplant Surgery, Ludwig Maximilians University of Munich, Munich, Germany; ^5^ Faculty of Bioengineering and Bioinformatics, Lomonosov Moscow State University, Moscow, Russia; ^6^ Belozersky Institute of Physico-Chemical Biology, Lomonosov Moscow State University, Moscow, Russia; ^7^ Scientific Center for Translation Medicine, Sirius University of Science and Technology, Sochi, Russia; ^8^ Institute of Translational Medicine and Biotechnology, Sechenov First Moscow State Medical University, Moscow, Russia; ^9^ Regenerative and Restorative Medicine Research Center (REMER), Research Institute for Health Sciences and Technologies (SABITA), Istanbul Medipol University, Istanbul, Türkiye

**Keywords:** acute myeloid leukemia, TIM-3, immunometabolism, glucose metabolism, lipid metabolism, galectin-9

## Abstract

**Introduction:**

T-cell immunoglobulin and mucin domain-3 (TIM-3) is a transmembrane molecule first identified as an immunoregulator. This molecule is also expressed on leukemic cells in acute myeloid leukemia and master cell survival and proliferation. In this study, we aimed to explore the effect of TIM-3 interaction with its ligand galectin-9 (Gal-9) on glucose and lipid metabolism in AML cell lines.

**Methods:**

HL-60 and THP-1 cell lines, representing M3 and M5 AML subtypes, respectively, were cultured under appropriate conditions. The expression of TIM-3 on the cell surface was ascertained by flow cytometric assay. We used real-time PCR to examine the mRNA expression of GLUT-1, HK-2, PFKFB-3, G6PD, ACC-1, ATGL, and CPT-1A; colorimetric assays to measure the concentration of glucose, lactate, GSH, and the enzymatic activity of G6PD; MTT assay to determine cellular proliferation; and gas chromatography–mass spectrometry (GC-MS) to designate FFAs.

**Results:**

We observed the significant upregulated expression of *GLUT-1*, *HK-2*, *PFKFB-3*, *ACC-1*, *CPT-1A*, and *G6PD* and the enzymatic activity of G6PD in a time-dependent manner in the presence of Gal-9 compared to the PMA and control groups in both HL-60 and THP-1 cell lines (*p* > 0.05). Moreover, the elevation of extracellular free fatty acids, glucose consumption, lactate release, the concentration of cellular glutathione (GSH) and cell proliferation were significantly higher in the presence of Gal-9 compared to the PMA and control groups in both cell lines (p < 0.05).

**Conclusion:**

TIM-3/Gal-9 ligation on AML cell lines results in aerobic glycolysis and altered lipid metabolism and also protects cells from oxidative stress, all in favor of leukemic cell survival and proliferation.

## 1 Introduction

Acute myeloid leukemia (AML) is defined by increased proliferative, undifferentiated, and non-functional hematopoietic cells in peripheral blood and/or bone marrow (1). This malignancy employs metabolic reprogramming as one of the key mechanisms to break through homeostasis. AML cells are highly reliant on aerobic glycolysis, characterized by excessive glucose consumption and lactate release. This glycolytic pattern encompasses the upregulation of glucose transporter-1 (GLUT-1) and two rate-limiting enzymes in this pathway: hexokinase-2 (HK-2) and 6-phosphofructo-2-kinase/fructose-2,6-bisphosphatase-3 (PFKFB-3). Several reports claim that the overexpression of GLUT-1 and HK-2 may be relevant to chemoresistance in AML patients (2–11). Also, the overexpression of glucose 6-phosphate dehydrogenase (G6PD), the rate-limiting enzyme in the pentose phosphate pathway (PPP), and the sensitivity of AML cells to its inhibition have been reported previously (12, 13). In line with G6PD, high levels of glutathione (GSH), to protect cells from oxidative stress, are expected in AML cells; therefore, hampering GSH production is considered for therapy (14, 15). Several studies reveal the dependence of AML cells, particularly leukemic stem cells (LSCs), on fatty acid oxidation (FAO) (16). This dependency may be due to not only providing energy but also the high capability of FAO in adjusting oxidative stress, even preventing anoikis (a kind of programmed cell death as a consequence of cell detachment from the extracellular matrix), as described in other malignancies (17–19). In this regard, the overexpression of carnitine palmitoyl transferase-1A (CPT-1A), the rate-limiting enzyme of FAO, has been reported previously in AML cells and is considered a target for therapy (20, 21). Moreover, abnormal lipogenesis in various cancers and hematologic malignancies are observed (22–24). The elevation of acetyl CoA carboxylase (ACC-1; the rate-limiting enzyme in lipogenesis) is considered a cancer hallmark in line with fatty acid synthase (FASN; another enzyme in lipid biosynthesis) in previous reports (22, 23, 25), though ACC-1 is not completely studied in AML and some data may be controversial (26). Since metabolic reprogramming in AML is a dynamic object of interest and several therapies are based on metabolic inhibitors (27), checking over the basics of these alterations, and particularly, identifying the upstream molecules that manipulate metabolic pathways in AML should be carried out to determine the role of leukemia-related markers.

More than a decade ago, T-cell immunoglobulin and mucin domain-3 (TIM-3), previously known as an immunoregulator, was identified on AML cells (28, 29). Rapidly, several studies were conducted to investigate the role of this molecule on leukemic cells. Galectin-9 (Gal-9), a β-galactoside binding lectin, was declared as its natural ligand in AML, as it binds to the IgV domain of TIM-3 and induces the phosphorylation of two tyrosine residues in the cytoplasmic tail of TIM-3 (30, 31). This leads to the activation of extracellular signal-regulated-kinase (ERK) as well as phosphoinositide 3-kinase/Protein kinase B/mammalian Target of Rapamycin (PI3K/Akt/mTOR) signaling pathway, which finally results in the activation and/or production of β-catenin, nuclear factor kappa B (NF-κB), vascular endothelial growth factor (VEGF), hypoxia-inducible factor-1 subunit alpha (HIF-1α), and tumor necrosis factor-alpha (TNF-α) (30, 32, 33). Therefore, TIM-3 is recognized as a promising target for immunotherapy and monoclonal anti-TIM-3 antibodies are currently under evaluation in several clinical trials for AML and high-risk MDS (myelodysplastic syndrome) in combination with cytotoxic therapies (NCT04266301, NCT04150029, NCT04823624, NCT03946670, NCT04878432, NCT03940352, and NCT03066648).

Currently, there are a few studies about the possible role of TIM-3 in metabolic reprogramming. A study on Jurkat T cells and another study on a macrophage cell line (RAW 264.7 cells) show that TIM-3 has a negative influence on glycolysis through downregulating GLUT-1 and HK-2 in these cells, respectively. Whether TIM-3 can affect other cells and other metabolic pathways, and in what directions, has not yet been assessed. Based on these data, we aimed to investigate the role of TIM-3 on metabolic reprogramming in AML cells by using two AML cell lines, HL-60 and THP-1, representing M3 and M5 AML subtypes, respectively^1^.

## 2 Materials and methods

### 2.1 Cell culture

HL-60 and THP-1 cell lines were obtained from Pasteur Institute (Tehran, Iran), cultured in Roswell Park Memorial Institute Medium 1640 (RPMI; BIO-IDEA, Iran) enriched with 10% and 15% fetal bovine serum (FBS; BIO-IDEA, Iran), respectively, in the presence of 1% antibiotic [penicillin G (10,000 U/mL) and streptomycin (10,000 U/mL); BIO-IDEA, Iran]. Cells were incubated at 37°C with 5.2% CO_2_ and 98% humidity. For each treatment, 5 × 10^5^ cells were transmitted to each well of a 24-well plate with 1 mL of complete medium. For the phorbol 12-myristate 13-acetate (PMA) group, cells were incubated with 50 ng/mL PMA (PeproTech, USA) for 24 h, to increase TIM-3 expression on the cell surface and lead the ligand to interact with TIM-3, aside from its other receptors like VISTA or CD44 on leukemic cells. While AML cells are known to produce Gal-9 (though in few amounts *in vitro*) to sharpen the effect of TIM-3, we designed Gal-9 groups in which, after exchanging the medium of cells that were exposed to 50 ng/mL PMA for 24 h, 100 ng/mL Recombinant Human Gal-9 (*Escherichia coli* expressed, carrier-free) (BioLegend, USA) was added to the medium. Cells or their supernatant were harvested after 24 h, 48 h, and/or 72 h after treatment with Gal-9 for each test, as required.

### 2.2 Flow cytometry

Cell lines were stimulated with 50 ng of PMA (PeproTech, USA) final concentration, for 24 h. FITC anti-human CD366 [(TIM-3); clone: F38-2E2, isotype: Mouse IgG1, κ] (BioLegend, USA) was used to stain stimulated cells vs. control cells. FITC mouse IgG1 κ isotype antibody (clone: MOPC-21; BioLegend, USA) was used to guarantee the specificity of the flow cytometric results. Surface TIM-3 was measured using the FACSCalibur instrument (Becton Dickinson Bioscience, USA) and data were analyzed with FlowJo software (Becton Dickinson, USA).

### 2.3 Real-time qPCR

Extracted total RNA (Total RNA Extraction Kit; Parstous Biotechnology, Iran) was used for cDNA synthesis (AddScript cDNA Synthesis Kit; Addbio, Sweden) in Thermal Cycler (Bio-Rad, Singapore). This product was subjected to qPCR (RealQ Plus 2x Master MixGreen, High ROX; Amplicon, Denmark) for target genes with primers (synthesis: metabion, Germany) listed in **Table 1**, alongside the housekeeping gene, β-actin, performed using the Applied Biosystem Real-Time PCR System (USA).

**Table 1:** List of studied gene expressions and their specific primer sequences.

Gene symbol	Orientation	Primers sequences
**ACTB**	Forward	TTCGAGCAAGAGATGGCCA
	Reverse	CACAGGACTCCATGCCCAG
**GLUT-1**	Forward	CTGGCATCAACGCTGTCTTCTAT
	Reverse	GCTCGCTCCACCACAAAC
**HK-2**	Forward	AGCCTGGACGAGAGCATC
	Reverse	TCACCACAGCAACCACATC
**PFK**	Forward	GGGGACAAATTGCGGTTTTC
	Reverse	CCACAACTGTAGGGTCGTC
**G6PD**	Forward	ATCAGTCGGATACACACATATTC
	Reverse	CGGAACAGCCACCAGATG
**ACC-1**	Forward	TGAAGCCAAGATAATCCAGCAG
	Reverse	CAAGCCATCCACAATGTAAGC
**ATGL**	Forward	GCCCAAGCGGAGGATTAC
	Reverse	CAGCAAGCGGATGGTGAAG
**CPT-1A**	Forward	ACTCACATTCAGGCAGCAAG
	Reverse	ATGGTGTCTGTCTCCTCTCC

### 2.4 Glucose consumption and lactate release

Glucose consumption (PARS AZMUN, Iran) and lactate release (Audit, Iran) were established by subtracting the concentration of each metabolite in fresh media (C-RPMI with 10% and 15% FBS for HL-60 and THP-1 cell lines, respectively) from the final concentration in the cell culture supernatant of the control (Ctrl), PMA, and Gal-9 groups (Hitachi 917; Boehringer Mannheim, Germany). For each treatment, the initial seeding number was 5 × 10^5^ cells per mL medium with three replicates, cultured in a 24-well plate.

### 2.5 GSH concentration and enzymatic activity of G6PD

A total of 6 × 10^6^ cells from each cell line for each test were grown under indicated treatments (Ctrl, PMA, and Gal-9 groups) with three replicates in six-well plates. Harvested cells were subjected to GSH concentration measurement (Navand Salamat Co., Iran) and G6PD enzymatic activity (Gesan, Italy) according to the manufacturer’s protocol.

### 2.6 MTT Cell viability assay

Each cell line was cultured with 10^5^ cells per mL per well seeding number for the Ctrl, PMA, and Gal-9 groups. MTT assay was performed according to the manufacturer’s protocol (DNA Biotech, Iran). Briefly, 10 µL of MTT solution (5 mg/mL in PBS buffer) was added to 90 µL of cell suspension containing 5 × 10^5^ cell/mL, and the plate was incubated at 37°C with 5.2% CO_2_ and 98% humidity for 3 h. Then, the resulting formazan crystals were dissolved by adding 100 µL of detergent reagent followed by gentle mixing in a shaker. Optical density was measured at 570 nm wavelength with a background absorbance of 690 nm (MPR4+, Hiperion, Germany).

### 2.7 Gas chromatography–mass spectrometry

Cell culture supernatant was collected from the Ctrl, PMA, and Gal-9 groups of each cell line and lyophilized by freeze dryer (DorsaTech, Iran) for 24 h. Ultrasonication for 90 minutes after adding 3 ml of N-hexane (Pars Chemie, Iran) to each sample helped the dissolving of lipids in hexane. This procedure was performed twice, and each time, the supernatant was collected after centrifugation (3,000 rpm, 5 min) and air dried completely. After workup, 50 µL of HPLC-grade chloroform (Neutron Pharmacochemical Co, Iran) was added to each dried sample, and 1 µl was injected into the GC mass system (Agilent US94324233, USA) on a capillary column (HP-5; 30 m × 0.25 mm, 0.25 μm) in a splitless mode with the column temperature program: 100°C (H = 2) @ R 12°C to 210°C (H = 0) @ R 3°C to 250°C (H = 5) with a mass ionization voltage of 30 eV. The MSD ChemStation was used as the operating software. Identification was done using Kovats retention index, NIST and Wiley275.L libraries, and spike with standards (methyl myristate; *t* = 17.6, methyl palmitate; *t* = 19.1, methyl oleate; t = 19.4, methyl stearate). The Kovats retention index was calculated according to the retention times of normal alkanes (C9–C24) with the same oven temperature program.

### 2.8 Statistical analysis

Every experiment was performed in triplicate. Statistical analysis was conducted using GraphPad Prism 9.4.1 and SPSS 26.0. To evaluate the assumption of normality, the Kolmogorov–Smirnov test was performed. Therefore, one-way ANOVA, a parametric method, was used to compare the differences, and a *p*-value of < 0.05 was considered a statistically significant result.

## 3 Results

### 3.1 The expression of TIM-3 on AML cell lines was elevated following stimulation with PMA

**Figure 1:**
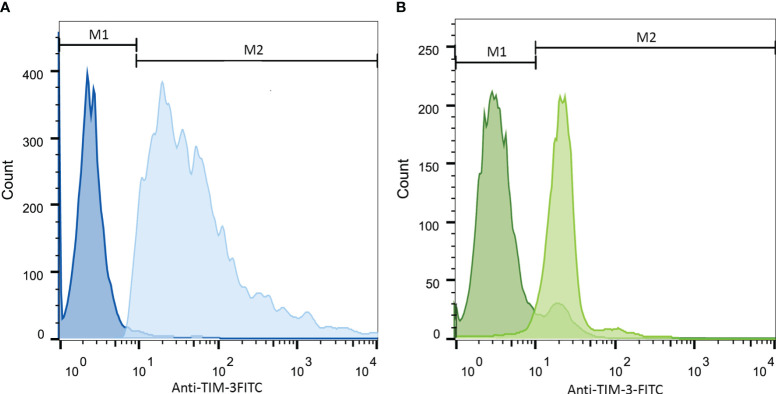
Increase in TIM-3 expression in HL-60 **(A)** and THP-1 **(B)** cell lines upon 24-h stimulation with PMA. Cells were incubated with 50 ng/mL/5 × 10^5^ cells of PMA for 24 h. Then, staining with anti-TIM-3 antibody conjugated with FITC was obtained and the expression of TIM-3 in the stimulated group compared to the control group was analyzed. Both HL-60 **(A)** and THP-1 cell **(B)** lines showed increase in TIM-3 expression from 4% ( ± 1.2) to 71% ( ± 1.9)and from 21% ( ± 2.5) to 84% ( ± 3.2), respectively. The darker color represents the control group.

As the first step, we examined the surface expression of TIM-3 on both cell lines following treatment with PMA within 72 h with flow cytometric assay and observed the maximum increase 24 h post-treatment. TIM-3 expression, 24 h after treatment, was increased from 4% to 71% and from 21% to 84% in HL-60 and THP-1 cell lines, respectively. Therefore, Gal-9 was added to the test wells 24 h after treatment with PMA (**Figure 1**). Also, as PMA itself can induce the activation of several transcription factors (34, 35), we used cells treated with PMA for 24 h as the second control group to compare Gal-9 groups with.

### 3.2 TIM-3/Gal-9 interaction stimulated glycolysis through upregulating the expression of *GLUT-1*, *HK-2*, and *PFKFB-3* in AML cell lines in a time-dependent manner

**Figure 2:**
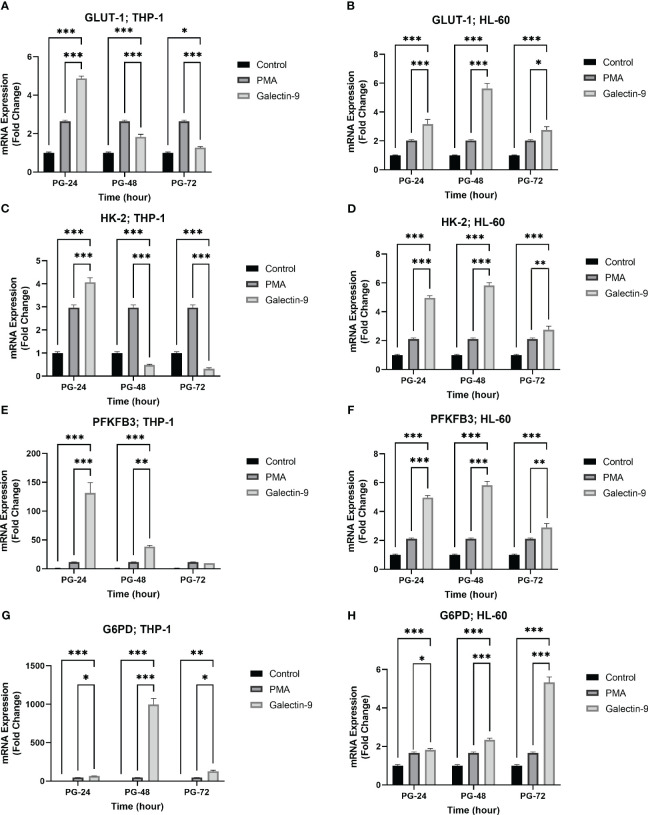
Increased mRNA expression of GLUT-1, HK-2, PFKFB-3, and G6PD in a time-dependent manner. The mRNA expression of GLUT-1, HK-2, and PFKFB-3 in the THP-1 cell line **(A, C, E)** was increased in the same time-dependent manner in the Gal-9 groups compared to the control and P-24 groups. A similar result was observed in the HL-60 cell line **(B, D, F)**. Also, G6PD showed increased expression at the transcriptional level in both cell lines compared to the control and PMA group **(G, H)** (*p < 0.05, **p < 0.01, ***p < 0.001).

To examine the role of TIM-3/Gal-9 interaction on glucose metabolism, we first analyzed the mRNA expression level of GLUT-1, HK-2, and PFKFB-3 in both cell lines. The same pattern of significant increase was observed for *GLUT-1*, *HK-2*, and *PFKFB-3* in both studied cell lines. In the HL-60 cell line, the transcript levels of *GLUT-1*, *HK-2*, and *PFKFB-3* were significantly increased after 24 h of treatment with Gal-9 and then decreased within 48 h and 72 h when compared to control and PMA groups (**Table 2**). In the THP-1 cell line, the expression of the same genes was significantly increased in all studied groups compared to the control and PMA groups, though the maximum increase was observed after 48 h of treatment with Gal-9. Also, the increase in mRNA expression level of *PFKFB-3* is not significant after 72 h of treatment with Gal-9 compared to the PMA and control groups (**Table 3**, **Figures 2A–F**).

**Table 2:** Significance of the differences after 24 h, 48 h, and 72 h compared to the control group and PMA group in HL-60 cells.

HL-60	Ctrl	PMA	24 h ^†^			48 h ^†^			72 h ^†^		
			Mean ± SD	vs. Ctrl	vs. PMA	Mean ± SD	vs. Ctrl	vs. PMA	Mean ± SD	vs. Ctrl	vs. PMA
***GLUT-1* ^‡^**	1.00 ± 0.05	2.01 ± 0.07	3.16 ± 0.32	*p <* 0.0001	*p =* 0.001	5.64 ± 0.34	*p <* 0.0001	*p <* 0.0001	2.75 ± 0.24	*p <* 0.0001	*p =* 0.02
***HK-2* ^‡^**	1.00 ± 0.06	2.11 ± 0.07	4.96 ± 0.15	*p <* 0.0001	*p <* 0.0001	5.82 ± 0.20	*p <* 0.0001	*p <* 0.0001	2.75 ± 0.25	*p <* 0.0001	*p =* 0.004
***PFKFB-3* ^‡^**	1.00 ± 0.05	2.11 ± 0.06	4.96 ± 0.15	*p <* 0.0001	*p <* 0.0001	5.82 ± 0.27	*p <* 0.0001	*p <* 0.0001	2.90 ± 0.27	*p <* 0.0001	*p =* 0.003
***ACC-1* ^‡^**	1.00 ± 0.07	0.48 ± 0.11	32.90 ± 1.27	*p <* 0.0001	*p <* 0.0001	10.09 ± 0.63	*p <* 0.0001	*p <* 0.0001	1.02 ± 0.05	*p =* 0.9	*p =* 0.8
***ATGL* ^‡^**	1.00 ± 0.04	2.11 ± 0.09	1.49 ± 0.10	*p =* 0.002	*p =* 0.0003	0.96 ± 0.17	*p =* 0.9	*p <* 0.0001	0.81 ± 0.09	*p =* 0.2	*p <* 0.0001
***CPT-1A* ^‡^**	1.00 ± 0.07	1.32 ± 0.09	10.79 ± 0.96	*p <* 0.0001	*p <* 0.0001	19.09 ± 0.72	*p <* 0.0001	*p <* 0.0001	36.13 ± 2.61	*p <* 0.0001	*p <* 0.0001
***G6PD* ^‡^**	1.00 ± 0.06	1.66 ± 0.05	1.82 ± 0.07	*p =* 0.0002	*p =* 0.6	2.34 ± 0.09	*p <* 0.0001	*p =* 0.0009	5.33 ± 0.28	*p <* 0.0001	*p <* 0.0001
**G6PD^ 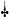 ^**	100.7 ± 2.5	106.0 ± 4.0	–			167.3 ± 8.3	*p <* 0.0001	*p <* 0.0001	–		
**Glucose^ 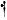 ^**	20.0 ± 0.5	28.0 ± 2.1	38.5 ± 2.3	*p =* 0.0006	*p =* 0.02	103.0 ± 6.5	*p <* 0.0001	*p <* 0.0001	113.5 ± 3.1	*p <* 0.0001	*p <* 0.0001
**Lactate^ 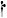 ^**	5.8 ± 0.2	7.0 ± 0.3	8.8 ± 0.4	*p =* 0.0003	*p =* 0.005	14.8 ± 0.5	*p <* 0.0001	*p <* 0.0001	15.2 ± 1.0	*p <* 0.0001	*p <* 0.0001
**GSH^ 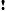 ^**	0.749 ± 0.031	1.324 ± 0.101	–			2.568 ± 0.106	*p <* 0.0001	*p <* 0.0001	–		
**MTT^ 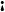 ^**	0.108 ± 0.008	0.130 ± 0.039	–			–			0.638 ± 0.119	*p =* 0.0008	*p =* 0.001

All tests are performed in triplicate [^†^: hours after treatment with Gal-9; ^‡^: mRNA expression (change fold); ^
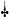
^: enzymatic activity of G6PD (mU/10^9^ cells); ^
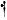
^: glucose consumption; ^
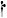
^: lactate release (mg/dL); ^
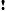
^: glutathione concentration (mM); ^
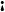
^: optical density]. ACC-1, Acetyl CoA carboxylase; ATGL, Adipose triglyceride lipase; CPT-1A, Carnitine palmitoyl transferase-1A; G6PD, Glucose 6-phosphate dehydrogenase; GLUT-1, Glucose transporter-1; GSH, Glutathione; HK-2, Hexokinase-2; PFKFB-3, 6-phosphofructo-2-kinase/fructose-2,6-bisphosphatase-3; MTT, 3-(4,5-dimethylthiazol-2-yl)-2,5-diphenyl-2H-tetrazolium bromide.

**Table 3:** Significance of the differences after 24 h, 48 h, and 72 h compared to the control group and PMA group in THP-1 cells.

THP-1	Ctrl	PMA	24 h ^†^			48 h ^†^			72 h ^†^		
			Mean ± SD	vs. Ctrl	vs. PMA	Mean ± SD	vs. Ctrl	vs. PMA	Mean ± SD	vs. Ctrl	vs. PMA
***GLUT-1* ^‡^**	1.00 ± 0.04	2.64 ± 0.06	4.87 ± 0.12	*p <* 0.0001	*p <* 0.0001	1.83 ± 0.14	*p =* 0.0001	*p <* 0.0001	1.27 ± 0.05	*p = 0.03*	*p <* 0.0001
***HK-2* ^‡^**	1.00 ± 0.06	2.97 ± 0.11	4.07 ± 0.19	*p <* 0.0001	*p <* 0.0001	0.48 ± 0.04	*p =* 0.0009	*p <* 0.0001	0.32 ± 0.04	*p <* 0.0001	*p <* 0.0001
***PFKFB-3* ^‡^**	1.00 ± 0.04	11.41 ± 0.51	131.6 ± 17.65	*p <* 0.0001	*p <* 0.0001	38.18 ± 2.38	*p =* 0.001	*p =* 0.01	9.58 ± 0.06	*p =* 0.6	*p =* 0.09
***ACC-1* ^‡^**	1.00 ± 0.04	4.96 ± 0.06	6.66 ± 0.17	*p <* 0.0001	*p <* 0.0001	2.23 ± 0.25	*p <* 0.0001	*p <* 0.0001	1.84 ± 0.21	*p =* 0.0009	*p <* 0.0001
***ATGL* ^‡^**	1.00 ± 0.05	8.37 ± 0.11	118.15 ± 5.56	*p <* 0.0001	*p <* 0.0001	20.18 ± 1.26	*p <* 0.0001	*p =* 0.001	0.75 ± 0.05	*p =* 0.9	*p =* 0.02
***CPT-1A* ^‡^**	1.00 ± 0.06	72.76 ± 0.70	118.6 ± 0.83	*p =* 0.02	*p =* 0.0005	2,048.00 ± 87.07	*p <* 0.0001	*p <* 0.0001	308.28 ± 5.00	*p =* 0.0001	*p =* 0.0002
***G6PD* ^‡^**	1.00 ± 0.04	45.53 ± 0.73	63.78 ± 2.73	*p =* 0.0004	*p =* 0.01		*p =* 0.0001	*p =* 0.0001	126.68 ± 14.47	*p =* 0.01	*p =* 0.03
**G6PD^ 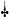 ^**	87.7 ± 7.2	141.0 ± 12.0	*-*			272.0 ± 11.5	*p <* 0.0001	*p <* 0.0001	–		
**Glucose^ 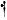 ^**	30.0 ± 0.5	55.0 ± 3.5	103.2 ± 4.0	*p <* 0.0001	*p <* 0.0001	121.0 ± 1.8	*p <* 0.0001	*p <* 0.0001	122.1 ± 1.2	*p <* 0.0001	*p <* 0.0001
**Lactate^ 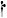 ^**	4.9 ± 0.1	5.6 ± 0.4	9.4 ± 0.3	*p <* 0.0001	*p <* 0.0001	11.2 ± 0.3	*p <* 0.0001	*p <* 0.0001	11.6 ± 0.7	*p <* 0.0001	*p <* 0.0001
**GSH^ 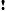 ^**	1.035 ± 0.208	1.492 ± 0.095	–			3.252 ± 0.203	*p <* 0.0001	*p <* 0.0001	–		
**MTT assay^ 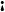 ^**	0.208 ± 0.004	0.259 ± 0.005	–			–			0.293 ± 0.004	*p <* 0.0001	*p =* 0.0002

Table legend: All tests are performed in triplicate [^†^: hours after treatment with Gal-9; ^‡^: mRNA expression (change fold); ^
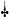
^: enzymatic activity of G6PD (mU/10^9^ cells); ^
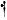
^: glucose consumption; ^
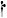
^: lactate release (mg/dL); ^
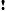
^: glutathione concentration (mM); ^
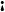
^: optical density].

ACC-1, Acetyl CoA carboxylase; ATGL, Adipose triglyceride lipase; CPT-1A, Carnitine palmitoyl transferase-1A; G6PD, Glucose 6-phosphate dehydrogenase; GLUT-1, Glucose transporter-1; GSH, Glutathione; HK-2, Hexokinase-2; PFKFB-3, 6-phosphofructo-2-kinase/fructose-2,6-bisphosphatase-3; MTT, 3-(4,5-dimethylthiazol-2-yl)-2,5-diphenyl-2H-tetrazolium bromide.

### 3.3 TIM-3/Gal-9 interaction results in an increase in glucose consumption and lactate release in AML cell lines

**Figure 3:**
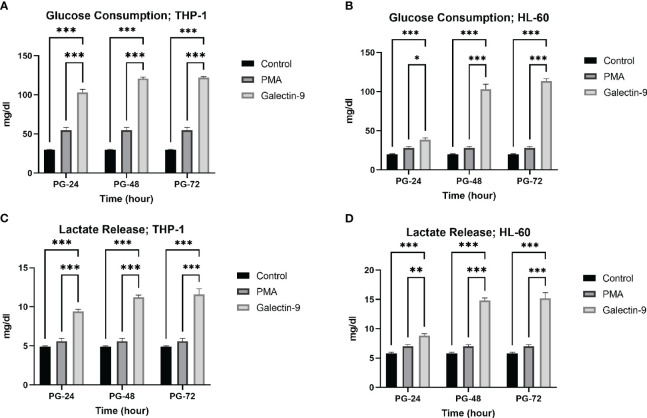
Increase in glucose consumption and lactate release. Glucose consumption **(A, B)** and lactate release **(C, D)** were increased in post-Gal-9 groups compared to the control and PMA group, and this increase is more significant over time in the HL-60 and THP-1 cell line (*p < 0.05, **p < 0.01, ***p < 0.001).

Next, we explored whether the upregulation of mRNA levels of *GLUT-1*, *HK-2*, and *PFKFB-3* could lead to a significant increase in glucose consumption (the substrate of glycolysis) and lactate release (as the final product of glycolysis). Interestingly, a significant rising trend was observed in glucose consumption and lactate release within 72 h of the experiment compared to the control and PMA groups in both cell lines (**Tables 2**, **3**, **Figure 3**).

### 3.4 TIM-3/Gal-9 interaction can alter lipid metabolism in AML cell lines in a time-dependent manner

**Figure 4:**
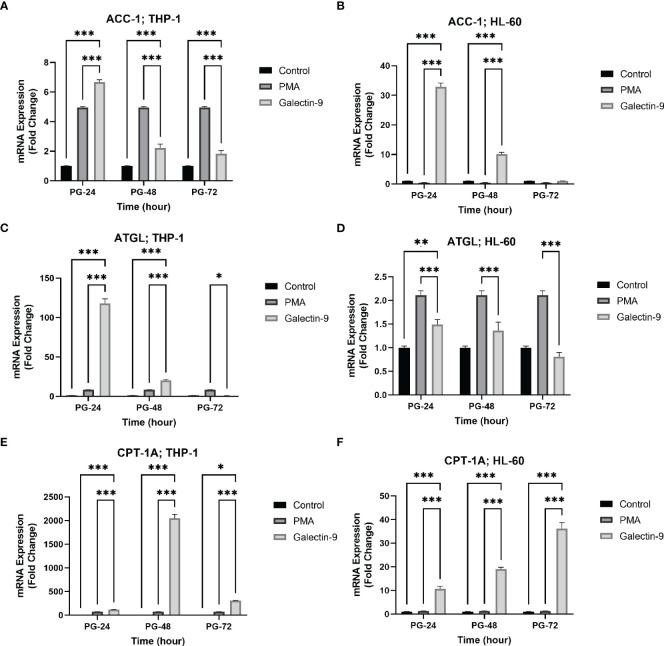
Increased mRNA expression of ACC-1, ATGL, and CPT-1A in a time-dependent manner. The mRNA expression of ACC-1 was increased in a time-dependent manner in the presence of Gal-9 compared to PMA and the control group in both cell lines **(A, B)**. The mRNA expression of ATGL was increased in Gal-9 groups compared to the PMA and control group in THP-1 **(C)**; however, the decrease and increase pattern of this enzyme in Gal-9 groups compared to the PMA and control groups in the HL-60 cell line seems to be impressed by PMA stimulation, as it decreased at 72 h **(D)**. The mRNA expression of CPT-1A **(E, F)** was increased in a time-dependent manner in Gal-9 groups compared to the PMA and control groups in both cell lines (*p < 0.05, **p < 0.01, ***p < 0.001).

To investigate the influence of TIM-3/Gal-9 interaction on lipid metabolism, the mRNA levels of *ACC*-1, *ATGL*, and *CPT-1A* in test groups compared to the control and PMA groups in both HL-60 and THP-1 cell lines were measured. The transcript level of *ACC-1* in the THP-1 cell line was significantly increased 24 h post-treatment with Gal-9 in comparison to the PMA and control groups (**Table 3**). Significant upregulation was also observed in the HL-60 cell line after 24 and 48 h of treatment with Gal-9 compared to the control group and PMA group, with the maximum elevation at 24 h post-treatment with Gal-9 (**Table 3**, **Figures 4A, B**).

The transcript level of *ATGL* in the THP-1 cell line was increased for 48 h, with the maximum increase in the first 24 h (**Table 3**). For the HL-60 cell line, 24 h after treatment, the mRNA level of *ATGL* was significantly increased in comparison to the control group but showed a significant decrease compared to the PMA group. Meanwhile, a significant decrease was also observed at 48 and 72 h post-treatment with Gal-9 compared to the PMA group (**Table 2**). Thus, TIM-3/Gal-9 interaction in the HL-60 cell line led to downregulation of *ATGL* expression (**Figures 4C, D**).

The mRNA level of *CPT-1A* was significantly increased in all test groups compared with the control and PMA group. The maximum elevation in the THP-1 cell line was observed at 48 h post-treatment with Gal-9, while the upregulated expression still followed a rising trend in HL-60 for 72 h (**Tables 2**, **3**, **Figures 4E, F**).

**Figure 5:**
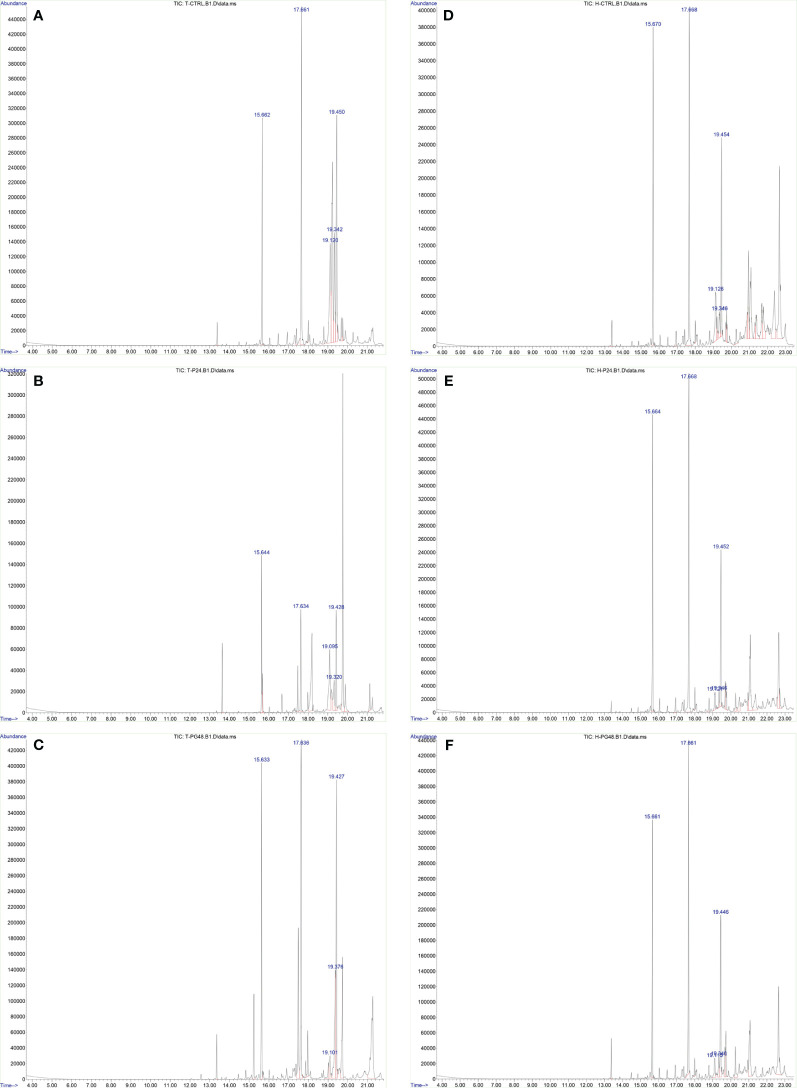
Increase of the percentage of FFAs 48 h after Gal-9 treatment. The relative abundance of FFAs (*t* = 15.6, methyl myristate; *t* = 17.6, methyl palmitate; *t* = 19.1, methyl oleate; *t* = 19.3, stearate; *t* = 19.4, methyl stearate) was increased 48 h after treatment with Gal-9 [THP-1: **(C)**, HL-60: **(F)**] compared to the PMA [THP-1: **(B)**, HL-60: **(E)**] and control [THP-1: **(A)**, HL-60: **(D)**].

Furthermore, we analyzed the relative abundance of free fatty acids in the supernatant of cell cultures 48 h after treatment with Gal-9, which showed the most significant alteration in comparison to the control and PMA group in both cell lines. The percentage of long-chain FFAs in the supernatant was significantly increased in the test group compared to the control and PMA group in the HL-60 cell line. However, in the THP-1 cell line, the percentage of long-chain fatty acids was significantly decreased compared to the control group but significantly increased compared to the PMA group as PMA led to a more significant decrease in FFAs than Gal9 (**Figure 5**, **Tables 4**, **5**).

**Table 4:** The results of gas chromatography–mass spectrometry (GC-MS) analysis of the supernatant of THP-1 cells in percentage.

THP-1	Ctrl (mean ± SD)	PMA (mean ± SD)	48 h after Gal-9 treatment		
			(mean ± SD)	vs. Ctrl	vs. PMA
**Methyl myristate**	10.5 ± 0.8	8.8 ± 0.7	12.7 ± 1.1	*p* = 0.04	*p* = 0.004
**Methyl palmitate**	1.1 ± 0.4	3.3 ± 0.6	9.0 ± 1.0	*p* < 0.0001	*p* < 0.0001
**Methyl oleate**	14.5 ± 0.7	12.7 ± 0.8	2.5 ± 0.7	*p* < 0.0001	*p* < 0.0001
**Stearate**	10.5 ± 0.9	5.4 ± 0.6	10.4 ± 1.1	*p* = 0.9	*p* = 0.001
**Methyl stearate**	17.0 ± 0.8	8.7 ± 0.6	16.1 ± 0.9	*p* = 0.3	*p* < 0.0001
**FFAs (sum)**	54.5 ± 2.4	43.4 ± 1.5	52.2 ± 1.9	*p* = 0.001	*p* < 0.0001

All tests are performed in triplicate.

Ctrl, control group; FFA, free fatty acid; PMA, phorbol 12-myristate 13-acetate.

**Table 5:** The results of GC-MS analysis of the supernatant of HL-60 cells in percentage.

HL-60	Ctrl (mean ± SD)	PMA (mean ± SD)	48 h Gal-9		
			(mean ± SD)	vs. Ctrl	vs. PMA
**Methyl myristate**	11.6 ± 0.6	17.4 ± 1.1	18.8 ± 1.2	*p* < 0.0001	*p* = 0.2
**Methyl palmitate**	15.0 ± 1.3	24.2 ± 1.0	22.0 ± 0.6	*p* < 0.0001	*p* = 0.08
**Methyl oleate**	5.3 ± 0.7	2.2 ± 0.4	3.0 ± 0.8	*p* = 0.01	*p* = 0.3
**Stearate**	2.2 ± 0.6	1.7 ± 0.6	2.9 ± 0.6	*p* = 0.3	*p* = 0.09
**Methyl stearate**	10.1 ± 0.6	12.6 ± 0.8	16.0 ± 0.7	*p* < 0.0001	*p* = 0.002
**FFAs (sum)**	44.2 ± 2.3	58.1 ± 1.1	62.7 ± 1.1	*p* < 0.0001	*p* = 0.02

All tests are performed in triplicate.

Ctrl, control group; FFA, free fatty acid; PMA, phorbol 12-myristate 13-acetate.

### 3.5 TIM-3/Gal-9 interaction can protect AML cell lines from oxidative stress by upregulation of G6PD expression and glutathione concentration

**Figure 6:**
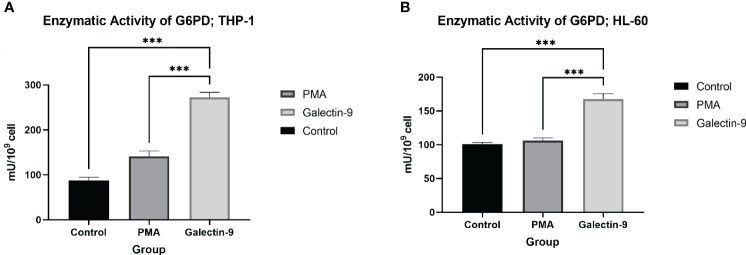
Increase in enzymatic activity of G6PD 48 h after Gal-9 treatment. The enzymatic activity of G6PD was elevated at 48 h after treatment with Gal-9 compared to the PMA and control group in both the THP-1 **(A)** and HL-60 **(B)** cell lines (***p < 0.001).

**Figure 7:**
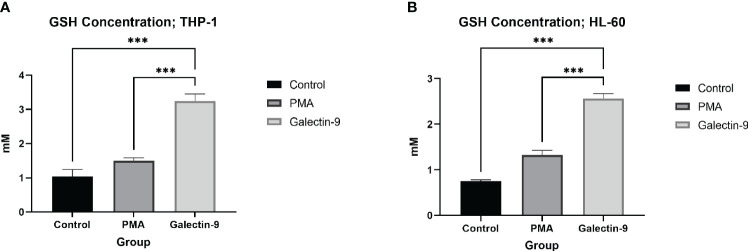
Elevation of GSH concentration 48 h after Gal-9 treatment. The concentration of GSH was increased at 48 h after treatment with Gal-9 compared to the PMA and control group in both the THP-1 **(A)** and HL-60 **(B)** cell lines (***p < 0.001).

In order to explore the potential role of TIM-3 in protecting AML cells from oxidative stress, we analyzed the mRNA level and enzymatic activity of G6PD and the concentration of GSH in test groups in comparison to the control and PMA groups. We observed significant elevation in *G6PD* expression after 24 h of treatment with Gal-9 compared to the control group and after 48 and 72 h of treatment with Gal-9 compared to the control and PMA group in both cell lines. The maximum increase was observed at 48 h after treatment with Gal-9 in THP-1, while the rising trend of *G6PD* mRNA level continued for 72 h (**Figures 2G, H**). Analysis of the enzymatic activity of G6PD and the GSH concentration showed a significant increase at 48 h post-treatment with Gal-9 in comparison to the control and PMA group in both HL-60 and THP-1 cell lines (**Tables 2**, **3**, **Figures 6**, **7**).

### 3.6 TIM-3/Gal-9 interaction promotes cell proliferation in AML cell lines

**Figure 8:**
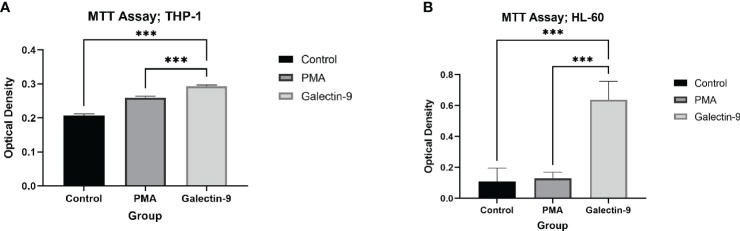
Increase in proliferation. The measured optical density in the MTT test, as a marker of proliferation and/or survival was elevated at 72 h after treatment with Gal-9 compared to the PMA and control group in both the THP-1 **(A)** and HL-60 **(B)** cell lines (***p < 0.001).

In order to conclude the effects of TIM-3/Gal-9 interaction on AML cell lines, we performed an MTT test on cells 72 h after treatment with Gal-9 in comparison to the PMA and control group. Cell proliferation in the Gal-9 group was significantly higher than that of the PMA and control group in both HL-60 and THP-1 cell lines (**Tables 2**, **3**, **Figure 8**).

## 4 Discussion

Several therapeutic approaches in AML are focused on metabolic pathways, as the proliferation and survival of leukemic cells are extremely dependent on metabolic reprogramming, especially the alterations in glucose and lipid metabolism (27). The abnormal expression of TIM-3 on leukemic cells and LSCs in AML is associated with cellular proliferation and survival, and might also be correlated with chemoresistance (36). Furthermore, there are two more recent studies on T cells and macrophages revealing that TIM-3 has a direct role in controlling glucose metabolism. As metabolic inhibitors are among the promising therapies for AML, and several clinical trials are exploring the effect of TIM-3 blockade in AML patients, we investigated the role of TIM-3 on glucose and lipid metabolism in two AML cell lines.

In the present study, we observed that TIM-3/Gal-9 interaction could consistently promote the elevation of glucose consumption and lactate release in both HL-60 and THP-1 cell lines. Analyzing the expression of *GLUT-1*, *HK-2*, and *PFKFB-3* showed that TIM-3 controls aerobic glycolysis by affecting the expression of these proteins. This is in line with the metabolic profile of glucose in AML, and the reduced proliferation in the presence of glycolysis inhibitors in leukemic cells (37–40). Apparently, the glycolytic pattern is associated with chemoresistance and high levels of mTORC1, and its activation following TIM-3/Gal-9 ligation has been reported previously (2, 3, 12, 40–42). Furthermore, HL-60 is known to be less glycolytic compared to other AML cell lines, including THP-1 (38); the lower expression of TIM-3 on the M-3 subtype of AML may be the cause. More interestingly, the metabolic profile was shown to be restricted in a time-dependent manner, as the increase in *GLUT-1*, *HK-2*, and *PFKFB-3* followed an uprising trend for 48 h in HL-60 and 24 h in THP-1 (the different pattern is probably related to the heterogeneous nature of AML). This is in line with the glycolytic pattern of several immune cells upon activation, in which the Warburg effect is considered a hallmark (activated Th1 cells, CD8+ cytotoxic T cells, natural killer cells, and myeloid cells like dendritic cells and M1 macrophages) (43–59). Lee et al. studied the relationship between TIM-3 expression and glucose metabolism in Jurkat T cells and reported that glucose consumption, lactate release, and GLUT-1 expression were decreased by TIM-3 overexpression and increased by TIM-3 knockout (60). Zhang et al. revealed that TIM-3 can prevent glycolysis in the RAW 264.7 macrophage cell line through downregulation of HK-2 expression (61). These are contrary to our results. However, considering the diverse roles of TIM-3 in different cells (62), this issue is not surprising anymore, though obviously the binary behavior of TIM-3 is still a mystery requiring more investigation to be figured out. Regarding lipid metabolism, a significant elevation in the expression of *ACC-1* in both cell lines, particularly in HL-60, within 24 h of treatment with Gal-9 was observed in our study. Though the expression declined at 48 and 72 h after Gal-9 treatment, it was no longer significant in the HL-60 cell line after 72 h of treatment. *De novo* FAS is not limited to liver and adipocytes, as this pathway is restored in malignancies and elevation of ACC-1 is considered as a cancer hallmark (22–25, 63–68). Also, switching to FAS and upregulated ACC-1 upon DC activation and M1 macrophage polarization has been reported previously (44–49, 51–53, 55, 59). The upregulation of FASN has been previously reported in the HL-60 cell line compared to healthy groups by Pizer et al. (68), though Southam et al. did not observe a significant increase in ACC-1 expression in the K562 (another AML cell line) or HL-60 cell line (26). We observed the significant upregulation of *ACC-*1 as a result of increased TIM-3 and in the presence of its ligand, Gal-9. On the other hand, the expression of ACC-1 in AML is not well studied yet. Considering the heterogeneous nature of this hematologic malignancy, examining the expression of FASN and analyzing intracellular fatty acids can help understand FAS in AML in the future.

The expression of *ATGL* in THP-1 was significantly increased within 48 h after treatment with Gal-9, with the most elevation within the first 24 h. In HL-60, the upregulation of *ATGL* within 24 and 48 h after Gal-9 treatment compared to the control group appears to be relevant to PMA, since the expression of *ATGL* is significantly decreased in comparison with the PMA group. Considering the heterogeneous nature of AML (69–71), which resulted in different expression patterns in THP-1 and HL-60 cell lines, and the sharp effect of PMA on the expression of *ATGL*, studying the role of TIM-3 on lipolysis requires a different study design, such as examining overexpressed or knocked-out cells.

The expression of *CPT-1A* was significantly elevated in both cell lines after treatment with Gal-9 in comparison to control groups; this increase was remarkably higher in THP-1. The dependence of AML cells, particularly LSCs, on high levels of FAO has been declared previously (16). This reliance is probably due to the high proficiency of FAO in balancing oxidative stress (17–19), which is the same as the physiological prolonged survival rate in immune memory cells (72, 73). Previous studies introduced CPT-1A as a target for therapy as its upregulation was associated with the worst clinical outcome in AML (20, 21). Also, Samudio et al. demonstrated that inhibition of CPT-1A results in enhanced sensitivity to cytotoxic agents in AML cells, probably based on the role of FAO in controlling mitochondrial permeability dependent on B-cell lymphoma 2 antagonist/killer (BAK) (74). After all, it can be concluded that in the HL-60 cell line, the fatty acid synthesis (FAS) pathway is activated at first, since the upregulation of ACC-1 was more significant in the first 24 h after the intervention and the FFA analysis showed a significant increase at 48 h after treatment with Gal-9. Later, as *ACC-1* declined, *CPT-1A* was upregulated in an increasing trend for 72 h post-treatment with Gal-9, showing that FAS is replaced with FAO. Meanwhile, the THP-1 cell line showed a sharp increase in *CPT-1A* within 24 and 48 h after treatment with Gal-9. Although this increased expression did not ascend in the last 24 h of our study, *CPT-1A* does not allow the slight upregulation of *ACC-1* in the first 2 days to elevate FFAs to the base level, as FFAs after 48 h treatment with Gal-9 significantly decreased compared to the control group. Overall, we observed that FAO was dominant as a result of TIM-3/Gal-9 interaction in THP-1 cells, even though it did not affect intracellular FFAs within 48 h after the intervention. Moreover, since we confirmed the upregulation of CPT-1A as a result of TIM-3/Gal-9 interaction in AML cell lines in this study, it might be interesting to examine whether TIM-3 is relevant to CD36 (an FA transporter)-positive LSCs, as these cells seem to represent an extraordinary metabolic profile with elevated FAO (75, 76).

Another interesting finding in our study was the elevation of FFAs (methyleC14-methylC18) in the supernatant from the cell culture after 48 h of treatment with Gal-9 compared to control groups. Moreover, the relative abundance of palmitate and oleate was increased after 48 h of treatment, but not significantly. To date, there are no reports on the FFAs that AML cells produce and release to the cell niche and we cannot be sure whether this incident occurs *in vivo* or whether it was observed due to *in vitro* conditions. A rational question concerning the significant elevation of the relative abundance of FFAs in the supernatant of cell culture is “For what purpose do leukemic cells produce them?”. Reviewing current knowledge on fatty acids, palmitate is one of the agonists of toll-like receptors; therefore, it can actively promote immune response (77). While the production of oleate can save cells from harmful saturated fatty acids (26), oleate might be able to show anti-inflammatory roles in some contexts (78). However, we could not measure the concentration of each free fatty acid or fatty acid methyl ester, and considering their probable role in preventing immune response, more study in this area is required. Furthermore, since *in vivo* LSCs directly interact with bone marrow niche and cells and not only affect them but also are affected by them, co-culturing AML cells with bone-marrow-derived adipocytes and assessing the effect of TIM-3/Gal-9 interaction on leukemic cells and adipocytes can be a subject of interest.

TIM-3/Gal-9 interaction can considerably help HL-60 and THP-1 cells to confront oxidative stress. The expression of *G6PD* was significantly elevated in both cell lines in the presence of Gal-9. The uprising trend was consistent within 72 h in HL-60, while the maximum increase in THP-1 was 48 h after treatment with Gal-9. Also, the upregulation observed in THP-1 cells was more considerable compared to HL-60 cells, referring to their different sources. Furthermore, we observed a significant increase in enzymatic activity of G6PD after 48 h of treatment with Gal-9 compared to control groups in both cell lines, attributed to the upregulation of this enzyme in the protein level as well. This is in line with previous studies on malignancies as the upregulation of G6PD in cancers has been discussed frequently (79). Also, this enzyme can be considered as a target for therapy, since inhibition of G6PD in AML results in cell cycle arrest or cell death and increased sensitivity in chemotherapeutic agents (80, 81). In the same context, we observed a significant elevation in the concentration of intracellular GSH. Previous studies showed that AML cells are dependent on extracellular cysteine to produce enough GSH to meet their excessive demand (14). In the same line, Pei et al. demonstrated that primary AML cells are intensively reliable on GSH as any disruption in GSH metabolism is not tolerable (15). Further investigations to reveal the role of GSH in AML relapse are the subject of interest of an ongoing project as well.

In conclusion, the present study revealed that TIM-3/Gal-9 interaction induces metabolic reprogramming in AML in a time-dependent manner, which is probably activated following the PI3K/Akt/mTOR and ERK signaling pathway. Stimulating the glucose and lipid metabolism in AML is probably one of the underlying mechanisms that adjust the role of TIM-3 in AML malignant cell’s survival and higher levels of proliferation, which made TIM-3 a promising target for therapy. This study is a step forward in defining the explicit role of TIM-3 in the pathogenesis of malignancies as well as the behavior of immune cells. Further investigation is required, not only for all the questions that remained unanswered in our limited study (such as the probable role of TIM-3 in lipolysis in the HL-60 cell line, the role of TIM-3 in other aspects of glucose and lipid metabolism, or the effect of TIM-3 on other metabolic pathways), but also to clarify the effect of TIM-3 on the metabolism of other immune cells, other AML subtypes, and primary AML cells, as well as in a co-culture system with other cells (to recreate the bone marrow microenvironment and cellular interactions) and *in vivo* models and clinical studies.

## Data availability statement

The original contributions presented in the study are included in the article/supplementary material. Further inquiries can be directed to the corresponding author.

## Ethics statement

Ethical approval was not required for the studies on humans in accordance with the local legislation and institutional requirements because only commercially available established cell lines were used. Ethical approval was not required for the studies on animals in accordance with the local legislation and institutional requirements because only commercially available established cell lines were used.

## Author contributions

MR: Conceptualization, Formal Analysis, Investigation, Writing – original draft, Writing – review & editing. MG: Investigation, Methodology, Writing – review & editing. BG: Investigation, Methodology, Writing – review & editing. AS: Conceptualization, Writing – review & editing. AB: Conceptualization, Writing – review & editing. AZ: Conceptualization, Writing – review & editing. MG-H: Conceptualization, Formal Analysis, Funding acquisition, Supervision, Writing – review & editing.

## Footnotes

1 American Type Culture Collection-THP-1 [Internet]. Available from: https://www.atcc.org/products/tib-202, American Type Culture Collection-HL-60 [Internet]. Available from: https://www.atcc.org/products/ccl-240
